# Vitamin D deficiency in western dwelling South Asian populations: an unrecognised epidemic

**DOI:** 10.1017/S0029665120000063

**Published:** 2020-08

**Authors:** Andrea L. Darling

**Affiliations:** Department of Nutritional Sciences, School of Biosciences and Medicine, Faculty of Health and Medical Sciences, University of Surrey, Guildford GU2 7XH, UK

**Keywords:** 25-Hydroxyvitamin D, Ethnicity, Epidemiology

## Abstract

Vitamin D deficiency (25-hydroxyvitamin D; 25(OH)D) is at epidemic proportions in western dwelling South Asian populations, including severe deficiency (<12⋅5 nmol/l) in 27–60% of individuals, depending on season. The paper aimed to review the literature concerning vitamin D concentrations in this population group. Research from the UK and Europe suggests a high prevalence of South Asians with 25(OH)D concentration <25 nmol/l, with most having a 25(OH)D concentration of <50 nmol/l. In Canada, South Asians appear to have a slightly higher 25(OH)D concentration. There are few studies from the United States, South Africa and Australasia. Reasons for vitamin D deficiency include low vitamin D intake, relatively high adiposity, sun exposure avoidance and wearing of a covered dress style for cultural reasons. Possible health effects of deficiency include bone diseases such as rickets and hypocalcaemia in children and osteomalacia in adults. Vitamin D deficiency may also increase the risk of other chronic diseases. Increased fortification of food items relevant to South Asian groups (e.g. chapatti flour), as well as increased use of vitamin D supplements may help reduce this epidemic. Introducing culturally acceptable ways of increasing skin exposure to the sun in South Asian women may also be beneficial but further research is needed to assess the effectiveness of different approaches. There may be a need for a South Asian specific vitamin D dietary intake guideline in western countries. To conclude, vitamin D deficiency is epidemic in South Asians living in western countries and there is a clear need for urgent public health action.

Contrary to its name, vitamin D is not a vitamin (‘vital amine’), but it is a pro-hormone, our main source being skin production under the action of sunlight exposure. Smaller amounts of vitamin D are also obtained from the diet. Aside from its classical actions in calcium metabolism and bone health, vitamin D has known immunomodulatory and anti-proliferative functions, affecting immune and cellular health. Vitamin D deficiency has therefore been associated with a wide number of health conditions^(1)^ alongside its classic role in preventing the bone diseases of rickets and osteomalacia. This means it is important for public health providers to prevent vitamin D deficiency. However, globally we have a vitamin D crisis. This is as expected in countries that are nearer to the Earth's poles, whereby we cannot make vitamin D in our skin for part of the year, but also, unexpectedly, in counties nearer the equator that have sunny climates^(2)^.

It is known that vitamin D deficiency is particularly prevalent in some ethnically diverse populations, such as persons of Black, Middle Eastern and South Asian ancestry, in western countries^(3)^. However, until recently there has been a lack of research into 25-hydroxyvitamin D (25(OH)D) concentration in these groups. Western dwelling South Asians are culturally diverse, coming from across the large South Asian sub-continent. They may include populations of Bangladeshi, Indian and Sri Lankan, or Pakistani origin. They may be Hindu, Muslim or from other religions. The diets consumed by South Asians can be diverse, varying from strict vegan to meat-eating diets. Indeed, definitions and practice of vegetarianism and veganism may vary within South Asian populations, as well as changing when South Asians move to western countries.

Also, South Asians living in western countries may be first- or second-generation migrants and may be from more traditional or from more westernised families. First-generation migrants may have migrated from South Asia itself or from diaspora (e.g. East or South Africa). On average, South Asians living in western countries are more likely than White Europeans to live in the most deprived neighbourhoods. However, this is likely to vary by country and specific South Asian groups studied. For example in the UK, 28–31 % of Bangladeshis and Pakistani populations tend to live in the poorest neighbourhood decile compared with only 8–9 % of Indian and White populations^(4)^. Similarly, in the UK 31 % of Indians compared with 16 % of Bangladeshi and Pakistani individuals, and 20 % of White individuals, are classified as having a professional occupation^(5)^. These social differences are likely to influence diet, health and lifestyle in these populations.

This review summarises the 25(OH)D concentration in South Asians dwelling in all countries with a western culture, and concludes that severe vitamin D deficiency is highly prevalent, suggesting a little recognised epidemic, with urgent public health intervention required. In terms of review methodology, studies that do not provide specific data for South Asians were not included in this review (e.g. those that gave results for mixed Asian groups, for example including East Asians as well as South Asians and did not show separate data for the South Asian subset). Reported concentration of 25(OH)D in this review includes both plasma and serum measurements and in the interest of brevity these will not be differentiated within the text. Finally, vitamin D intake is reported as combined vitamins D_2_ and D_3_ unless otherwise stated.

## Epidemiology of 25-hydroxyvitamin D deficiency in South Asians in western countries

This section will discuss individual studies assessing 25(OH)D concentration in South Asian populations living in the UK and mainland Europe.

### United Kingdom

The majority of Europe's South Asian population lives in the UK, with 2–19 % of the UK population comprising individuals of South Asian ethnicity, depending on region^(6)^. Research shows that vitamin D deficiency (25(OH)D concentration <25 nmol/l) is common in UK dwelling South Asians in both adults and children. As illustrated in Table 1, for studies that indicated a specific season of measurement, ranges of 25(OH)D concentration included: summer 23–45 nmol/l; autumn 16–21 nmol/l; winter 10–20 nmol/l and spring 13–22 nmol/l. In studies not stating season of measurement, or who included multiple seasons in one estimate, 25(OH)D concentration was 11–42 nmol/l. These relatively broad ranges suggest a degree of between study variability, likely due to differing UK geographical areas and South Asian sub-populations (e.g. by religion and country of origin) studied. Of note, only three studies assessed vitamin D status in South Asian children, and these studies were conducted 20–40 years ago. The Rochdale study (1982) showed a 25(OH)D concentration of 19–25 nmol/l^(7)^. The Hunt *et al*. (1976) study found a mean 25(OH)D concentration of 16 nmol/l in 14-year-old girls^(8)^. Results of a Department of Health National Feeding Survey (1994–1996) showed that 34 % of Pakistani, 25 % of Indian and 20 % of Bangladeshi children had 25(OH)D concentration <25 nmol/l^(9)^.

**Table 1.** Details of published studies assessing 25 hydroxy vitamin D (25(OH)D) concentration specifically in UK dwelling South Asians, including characteristics of studies and reported 25(OH)D concentrations

Author, year, study	United Kingdom City	Latitude (°N)	Participants	Season/months	Median/mean 25(OH)D (nmol/l)*			Data on % under 25(OH)D cut-offs
					Group	Median or mean	sd or IQR	
Brooke-Wavell *et al.*, 2008^(81)^	Loughborough	52⋅8	Postmenopausal women *n* 24 aged 55–65 years	Late summer	All	Mean: 33	sd: 13	None
Darling *et al.*, 2013, D-FINES I^(10)^	Guildford	51⋅2	Pre-menopausal women *n* 35 aged 20–55 years		All	Mean:	sd:	Summer 54 % <25 nmol/l Winter 81 % <25 nmol/l
				Summer		26	10	
				Autumn		21	12	
				Winter		20	11	
				Spring		22	11	
Farrar *et al.*, 2011^(48)^	Manchester	53⋅5	*n* 15 20–60 year old adults, male and female	Winter	All	Median: 16	min, max: 7, 28	27 % <12⋅5 nmol/l 100 % <50 nmol/l
Finch *et al.*, 1992^(13)^	London	51⋅5	*n* 297 40 years old, male and female			Mean:	95 % CI	–
				Winter	NV	19	(17–22)	
				Summer	NV	45	(36–56)	
				Winter	V	10	(7–13)	
				Summer	V	27	(20–40)	
Hamson *et al.*, 2002^(82)^	Leicester	52⋅6	*n* 113 20–40 years old, male and female, Gujurati	–		Mean	Not given	50–60 % <12⋅5 nmol/l
					Female	23		
					Male	25 (Those over 12⋅5 nmol/l only: 50–60 % of subjects had <12⋅5 nmol/l)		
Hunt *et al.*, 1976^(8)^	London and Reading	51⋅5	*n* 52 45 years old, *n* 29 14 year old girls, male and female	Autumn		Mean:	sd:	28 % <8⋅7 nmol/l (all subjects)
					Men	16	11	
					Women	16	13	
					Girls	16	8	
Kift *et al.*, 2013^(83)^	Manchester	53⋅5	*n* 87 20–60 years old, male and female			Median:	IQR:	40 % <12⋅5 nmol/l winter 10 % <12⋅5 nmol/l summer 98 % <50 nmol/l winter 93 % <50 nmol/l summer
				Winter	All	15	10–20	
				Summer	All	23	17–33	
Lawson and Thomas, 1999^(9)^	Country wide (Department of Health Infant feeding survey)	–	*n* 618 South Asian children (Bangladeshi Indian, Pakistani)	–		Mean:	sd:	<25 nmol/l:
					Bangladeshi	42	21	20 %
					Indian	42	23	25 %
					Pakistani	36	20	34 %
Lowe *et al.*, 2010^(84)^	Blackburn	53⋅7	*n* 66 Pakistani postmenopausal women median age 55 years	May to September	All	Median: 11	IQR: 12	85 % <25 and 97 % <50 (11 % below limit of detection)
Macdonald *et al.*, 2010, D-FINES I^(12)^	Surrey data for South Asians	51⋅2	*n* 35 Postmenopausal women mean age 61 years			Median:	IQR:	<25 nmol/l Summer 51 % Autumn 75 % Winter 65 % Spring 66 %
				Summer	All	24	16	
				Winter	All	17	16	
Mavroeidi *et al.*, 2010 D-FINES I^(11)^	Surrey data for South Asians	51⋅2	*n* 35 Postmenopausal women mean age 61 years			Mean:	sd:	
				Winter/spring	All	23	11	–
				Summer/autumn	All	26	11	–
Patel *et al.*, 2013^(85)^	Birmingham	52⋅5	*n* 1122 South Asian males and females, mean age 58 years old	–		–	–	Females: 44 % <μg/l Males: 43 % <6 μg/l
Roy *et al.*, 2007^(86)^	Manchester	52⋅5	*n* 78 18–36 year old Pakistani women	Not stated	All	Mean: 20	sd: 11	94 % <38 nmol/l 26 % <13 nmol/l
Serhan and Holland 2002^(87)^	Wolverhampton	52⋅6	*n* 110 Indian men and women mean age 51 years, rheumatology patients	Not stated	All	Mean: 3⋅3–4⋅5 μg/l	sd: 2⋅6–2⋅1 μg/l	–
Shaunak *et al.*, 1985^(88)^	No area specified	–	*n* 120 21–75 years old, Hindu males and females	Spring	All	Mean: 21 (1)	sem: 1	22 % ≤10 nmol/l 57 % ≤20 nmol/l
Solanki *et al.*, 1995^(89)^	Birmingham	52⋅5	*n* 54 males and females, 20–91 years old	Spring	Older (>65 years): Females Males Younger (<65 years) Females Males	Mean(sd)	sd:	Older 57 % <15 nmol/l Younger 41 % <15 nmol/l
						23	20	
						13	9	
						21	7	
						16	8	
Stephens *et al.*, 1982^(7)^	Rochdale	53⋅6	*n* 159 adults and *n* 103 children (aged 5–18 years)	Not stated	Children Boys Girls Adults	Mean in 1980:	sd in 1980:	In 1980: Men: 35 % <13 nmol/l Women: 33 % <13 nmol/l Boys: 22 % <13 nmol/l Girls: 33 % <13 nmol/l
						25	17	
						19	10	
						20	15	
Ward *et al.*, 2007^(90)^	Manchester	53⋅5	*n* 95, mean age 29 years, premenopausal females	Not stated	All	Mean: 20	sd: 12	–

D-FINES, Diet, Food Intake and Exposure to Sunlight in Southern England study; V, vegetarian; NV, non-vegetarian.

* Unless otherwise stated.

Looking at specific UK geographical areas, 25(OH)D concentration appears to be highly variable within regions, although deficiency (<25 nmol/l) is highly prevalent in all areas. Three studies were conducted in Southern England (latitude 51°N); namely the Diet, Food Intake and Exposure to Sunlight in Southern England (D-FINES) study (women of mainly Pakistani origin)^(10–12)^, a study by Finch *et al*.^(13)^ and a study by Hunt *et al*.^(8)^. These studies found a mean or median 25(OH)D concentration of 26–45 nmol/l in summer, 16–21 nmol/l in autumn, 10–20 nmol/l in winter and 22 nmol/l in spring^(8,10–13)^. The remaining UK studies were from the Midlands or the North of England (latitude 52–53°N) and found a mean or median 25(OH)D concentration of 23–33 nmol/l in summer, 15–16 nmol/l in winter and 13–23 nmol/l in spring. For those with no season stated or mixed seasons, 25(OH)D varied from 11 to 25 nmol/l.

Therefore, there does not seem to be a systematic difference in 25(OH)D by latitude within the UK, at least within the latitude band of 51–53°N, despite the fact that the North of the UK is generally colder, cloudier and wetter than the South of the UK, with a lower UVB index. Any true latitude or climate differences may be obscured by the varying methods and populations used between the studies in different areas. The lack of a latitude difference could also be explained by the lower sunlight exposure and covered dress style of the South Asian populations which may make latitude and climatic differences less impactful on 25(OHD) concentration than would be the case in groups that expose plentiful skin to the sun and spend a lot of time in direct sunlight. More research is required in the far North of the UK (Scotland) to see if South Asians living in these areas have lower 25(OH)D concentrations as would be expected by the high latitude, cold climate and the slightly shorter photoperiod for which vitamin D can be made during the year. Also, there are no epidemiological studies, to the author's knowledge, assessing 25(OH)D concentration in South Asians dwelling in Northern Ireland, Scotland or Wales.

### Mainland Europe

Vitamin D deficiency in Europe is pandemic^(14)^. As in the UK, vitamin D deficiency in South Asians living in mainland Europe is highly prevalent. Most research has been undertaken in the Nordic countries. In terms of Norway, most studies have assessed Pakistani or Sri Lankan groups. Studies of South Asian adult have found that 75–91 % have 25(OH)D <50 nmol/l and 35–65 % have 25(OH)D <25 nmol/l^(15–18)^. Studies have also found a mean 25(OH)D concentration of 23–32 nmol/l^(17–20)^. In terms of pregnancy, a study of pregnant Pakistani women in Oslo found that at 18 weeks of gestation, median 25(OH)D was 19 nmol/l and 83 % of the women had 25(OH)D <30 nmol/l^(21)^.

In Finland, a study of Bangladeshi women (20–48 years old) found a mean 25(OH)D concentration of 43 nmol/l, with 71 % having 25(OH)D concentration of 25–50 nmol/l, 24 % having 51–74 nmol/l, and only 6 % having >75 nmol/l^(22)^. Interestingly, despite the far northern latitude and cold climate, no women had a 25(OH)D concentration <25 nmol/l^(22)^. It is unclear as to why this is the case, as only 10 % of the Bangladeshis used a vitamin D supplement. It is unlikely to be due to the very successful Finnish milk fortification programme, which has enhanced the 25(OH)D concentration of the Finnish population to a large degree, as the Bangladeshi women in the study generally did not consume milk products^(22)^. One alternative explanation is a high consumption of oily fish (e.g. a large portion daily) which could explain why no individuals were <25 nmol/l, although this was not discussed in the paper. In Denmark, Anderson *et al*. found in a Pakistani population a median 25(OH)D concentration of 12 nmol/l in women, 21 nmol/l in men and 11 nmol/l in girls^(23)^. In contrast, a Danish randomised control trial by Gronborg *et al*. found a baseline 25(OH)D of 47 nmol/l in Pakistani women^(24)^. In the Netherlands, in a study of Surinamese South Asian individuals, 51 % had 25(OH)D <25 nmol/l^(25)^.

Finally, in terms of the Mediterranean countries, one Italian study found that 76 % of Indian pre-schoolers (median age 1⋅5 years) had 25(OH)D <75 nmol/l and 54 % had 25(OH)D <50 nmol/l^(26)^. In Greece, among Bangladeshi patients with diabetes, mean 25(OH)D over the year was 31 nmol/l, with 90 % <50 nmol/l, 8⋅6 % 50–75 nmol/l and 1⋅2 % >75 nmol/l^(27)^.

### North America

A significant number of South Asians live in North America, in both Canada and the United States (US) (Canada 5⋅0 % of population (1⋅9 million people^(28)^) *v.* US 1⋅5 % of population (5⋅4 million people^(29)^)) with two-thirds of Canadian-based South Asians living in the Vancouver and Toronto areas. In Canadian South Asians vitamin D deficiency appears to be less severe than in those living in Europe. For example, in studies of 20–79-year-old South Asians, mean 25(OH)D measurement has been found to be 42–50 nmol/l^(30–32)^. In studies reporting vitamin D cut-off points, 34–38 % had 25(OH)D <30 nmol/l^(32,33)^ and 44 % had 25(OH)D <50 nmol/l.^(33)^ Interestingly, in a study of older South Asians (>60 years old) in Greater Toronto, mean 25(OH)D was relatively high at 77 nmol/l. This is likely explained by the high vitamin D intake in this group at 28 μg daily, which must have come from supplement use^(34)^.

There are few studies assessing 25(OHD) concentration in US dwelling South Asians, but in a study of Californian middle-aged South Asian Indian adults the mean 25(OH)D concentration was 48 nmol/l^(35)^. In contrast, in the Northern US (Minnesota), young South Asian adults aged 20–40 years only had a mean 25(OH)D of 33 nmol/l^(36)^. Finally, in Oklahoma, results from the Asian Indian Diabetic Heart Study and the Sikh Diabetes Study showed that vitamin D deficiency was highly prevalent with 51 % of middle-aged women and 58 % of middle-aged men having a 25(OH)D concentration <30 nmol/l^(37)^.

### Other western countries

There is a lack of data on vitamin D concentration in South Asians dwelling in other western countries, but some research has been undertaken in New Zealand and South Africa. In a study set in Auckland, New Zealand, 84 % of South Asian women had 25(OH)D <50 nmol/l, with a median 25(OH)D concentration of 28 nmol/l^(38)^. In a study of 18–65-year-old South African Asian-Indians in Johannesburg^(39)^, mean 25(OH)D was 36 nmol/l in women and 45 nmol/l in men^(39)^, with overall 25(OH)D concentrations of about 65 nmol/l in winter, 40 nmol/l in spring, 60 nmol/l in summer and 80 nmol/l in autumn^(39)^. More research is required into the 25(OH)D concentration of South Asian groups in western countries outside of Europe and North America.

## Reasons for 25-hydroxy vitamin D deficiency in western dwelling South Asians

### Low dietary vitamin D

In terms of dietary sources, we consume vitamin D from foods such as oily fish, meat, eggs (vitamin D_3_) and mushrooms (vitamin D_2_). Few commonly consumed foods in the typical western diet contain significant amounts of vitamin D; therefore, the majority of vitamin D is gained from sunlight exposure. However, because people do not spend enough time in the sunlight, and there are concerns about increasing sun exposure due to skin cancer risks, most western countries have a recommended dietary intake of vitamin D. However, without consuming fortified foods this recommended intake is often difficult to achieve, due to few commonly consumed foods that are rich in vitamin D.

Currently, the vitamin D guidelines for multiple countries do not contain any specific guidelines for South Asian adults, for example the US Institute of Medicine^(40)^, the European Food Standards Agency^(41)^ or The UK Scientific Advisory Committee on Nutrition^(42)^. The recommended intake is the same as for all adults for that particular country. For example, the UK guideline for South Asian children (aged 4 years and over) and adults is the same as for the rest of the population in the UK (10 μg daily; as per the Scientific Advisory Committee on Nutrition Report^(42)^), likely due to a lack of published scientific evidence assessing whether this should be higher in South Asians.

There are little data assessing vitamin D intakes of South Asian populations in western countries, and with an under-representation of South Asians in national dietary surveys reliance is placed on data from research studies. Published research data suggest an average intake of between 1 and 3 μg daily in western dwelling South Asians. For example, recent data from the UK Biobank cohort (baseline data, 2006–2010, adults aged 40–69 years) showed that UK South Asians had a vitamin D intake of 1–3 μg daily, with 1 μg daily in Indians, 1⋅5 μg daily in Pakistanis and 3 μg daily in Bangladeshis^(43)^. Of note, the Indian and Pakistani intakes were lower than the average found in the UK National Diet and Nutrition Survey (1⋅8–3⋅2 μg daily)^(44)^. It is likely that the lower intake in Indians was partly due to vegetarianism and veganism, in that some of the richest sources of vitamin D in the diet is oily fish and eggs, and although meat is not a rich source of vitamin D, in meat consumers a relatively large daily consumption of meat could contribute a significant proportion of vitamin D intake.

The D-FINES I study (2006–2007) found a vitamin D intake of 1⋅6–2⋅2 μg daily, depending on season, in premenopausal South Asian women^(10)^. These women were majority Pakistani and so were mostly meat-eaters^(10)^. Kift *et al*. found similar intakes in adult South Asians in Manchester (UK) at 1⋅3 μg daily^(45)^ and George *et al*. found a vitamin D intake of 1⋅2 μg daily in South African Asian-Indians^(39)^. The 1970s UK Rochdale study found a vitamin D intake of 2⋅5–2⋅8 μg daily (depending on age and sex), which was similar to the White Europeans also assessed in their paper (2⋅1–3⋅6 μg daily)^(7)^.

In North America, vitamin D intakes appear to be slightly higher than in Europe. Vitamin D intake in the Ottawa study was 4⋅7 μg daily in White Europeans and 4⋅4 μg daily in South Asians^(31)^. In the US (Chicago), in Indian adolescents and young adults, dietary intake was 3⋅8–8⋅7 μg daily^(46)^. Finally, in Greater Toronto, mean vitamin D intake from food in older adults was 4 μg daily^(34)^ and in younger adults intake from combined food and supplements was 8 μg daily^(47)^.

Reasons for low intake of vitamin D in South Asian groups may include dietary preferences, religious or cultural dietary requirements, and cost and availability of foods that contain vitamin D. In terms of foods contributing to vitamin D intake, Farrar *et al*. reported that eggs and egg dishes were the biggest contributor to vitamin D intake in UK South Asians, followed by red meat and milk^(48)^. Of note, oily fish did not contribute significantly to vitamin D intake in their group of South Asian adults^(48)^. However, the sample size was very small so this may not be representative of South Asians in general in the UK. Similarly, in the D-FINES I study of postmenopausal and premenopausal women (data published in abstract form only^(49)^), flour, grains and starches contributed the most to intake, followed by meat and meat products. Therefore, the contribution to intake varies by study, again likely due to differences in sample size, population studied and methods used to assess vitamin D intake.

Vegan South Asians, as well as some vegetarian South Asians, may not consume oily fish or eggs, so rely on vegetarian sources of vitamin D, as well as vitamin D produced from sunlight on the skin. The culture of some traditionally non-vegetarian South Asian populations includes a traditional diet which contains a high proportion of oily fish, for example those of Bangladeshi heritage^(50)^. Bangladeshi individuals may find in western countries that oily fish from their traditional diet are not readily available in their local shops, unless they live in some areas such as London's Tower Hamlets where there is a widespread availability of Bangladeshi dietary staples^(51)^. Moreover, unfamiliarity with commonly consumed and cheap fish in western countries, such as sardines or mackerel, may mean Bangladeshis make less use of these fish sources^(52)^. In terms of finances, in other ethnic groups, research has shown that oily fish may be perceived as a relatively expensive food to purchase^(53)^, meaning less is likely to be consumed. Research has shown that meat consumption is higher in UK-based Bangladeshis compared with Bangladeshis living in Bangladesh^(52)^, but there is no data on whether this is concurrent with a reduced fish intake. Finally, it must be borne in mind that more westernised individuals may also not consume their traditional diet and so may choose western foods which may include little or no oily fish, eggs or other sources of vitamin D.

### Low usage of dietary supplements

Some research has assessed supplement use in western dwelling South Asians and has found that only a small proportion of individuals use a vitamin D containing supplement. In a South African study, usage of all vitamin D supplements was low in Asian-Indians, at only 15 % of individuals^(39)^. A recent study from the UK Biobank cohort (*n* 7753) found that 23 % of men and 39 % of women took a vitamin D containing supplement (either a multivitamin and mineral or a single vitamin D supplement, or both)^(43)^. Of note, these data were collected in 2006–2010 so do not reflect the usage of supplements subsequent to Public Health England (UK) suggesting a winter-time usage of vitamin D supplements in 2016^(42)^ for at-risk groups such as South Asians, although the previous UK Committee of Medical Aspects of Food Policy guidelines (1991)^(54)^ did also recommend 10 μg daily for at groups at risk of vitamin D deficiency. Unfortunately, the UK Biobank study could not assess cod liver oil consumption as the UK Biobank questions only asked whether fish oil supplements were consumed or not, not specific subtypes of fish oil. In some countries cod liver oil may represent a significant intake of vitamin D for some individuals. For example, in Norway, a study found that 5 % of Pakistani women used cod liver oil daily^(17)^.

Reasons for use or non-use of supplements may be South Asian specific or similar to other ethnic groups. UK-based focus group research suggested that South Asian women were very happy to take supplements, in contrast to White European women who reported being less keen to do so^(55)^. Interestingly, in another study South Asians reported preferring injections to oral supplement tablets, believing it was more powerful, although some felt that supplements were ‘unnatural’^(56)^. In the study by Lawson and Thomas, only 5 % of South Asian children were reported as being given the Department of Health's combined vitamins A, C and D drops^(9)^. Availability and choice in the UK of vitamin D supplements has increased rapidly in recent years so it is unclear whether vitamin D supplementation in UK dwelling South Asian children has now improved. Further research is required to assess the usage of vitamin D containing supplements in children in the UK and elsewhere, to see if campaigns and improved product availability have improved the usage of vitamin D supplements.

### Low sun exposure

Few research studies have assessed sun exposure in western dwelling South Asians but all have found it to be relatively low, with some qualitative research finding that South Asian adults report sun avoidance. For example, in the D-FINES I study, summer sun exposure was 2 standard erythemal doses daily, compared with 6 standard erythemal doses in White European women^(10)^. A study in Manchester (UK) found that South Asian adults spent a median of 63 min outdoors on a summer weekday and 80 min daily on a summer weekend day^(45)^. Sunscreen use was low, with <10 % using sunscreen in any season^(45)^.

In terms of qualitative research, a study of New Zealand-based South Asian adults found that they reported avoiding sunlight and spending a lot of time indoors, as well as being concerned about skin cancer and the strength of the sun^(38)^. This was not specific to Australasia as a qualitative study conducted in London (UK) found similar results in that South Asians reported not being keen on sun exposure, preferring to sit in the shade and citing concerns about skin cancer^(56)^.

Research suggests that, as with other ethnic groups, current sun exposure in South Asians is not sufficient to produce adequate vitamin D status. Recent research from Manchester, in the UK (latitude 53⋅5°N), suggests that casual sun exposure is unlikely to provide adequate vitamin D production in South Asian individuals^(48)^. This study used three times weekly UV radiation in an irradiation cabinet to simulate 15–90 min of noon-time sunlight exposure, exposing 35 % of skin. The exposure led to a rise of only 11 nmol/l over 6 weeks, whereby there was a plateau. A post-treatment average 25(OHD) of 28 nmol/l meant that many individuals were still <25 nmol/l^(48)^. Other UK research using artificial UV radiation to stimulate 25(OH)D production in South Asian women showed that the baseline level of 25(OH)D was important in determining a response to ultraviolet radiation in that the most deficient women at baseline showed the largest gain in 25(OH)D concentration post UV radiation exposure^(57)^. This suggests that the most deficient individuals have the most to gain from sunlight exposure, but it must be borne in mind that this was only a very small study.

### Obesity

The prevalence of overweight and obesity is high in western dwelling South Asians. For example, in the UK, data from the Active Lives survey in 2017–2018 showed that 57 % of South Asians over the age of 16 years are overweight or obese^(58)^. This is of concern as it is known that a higher BMI is associated with a lower 25(OH)D measurement^(59)^. It is unclear why this is the case but it has been suggested that it is possibly due to either adipose sequestering 25(OH)D, or due to a higher fluid volume in those with a larger body size (volumetric dilution), but recent research supports the volumetric dilution as the best explanation^(60)^. In the Asian Indian Diabetic Heart Study, those with BMI >27⋅5 kg/m^2^ had a statistically significantly lower 25(OH)D than those with BMI <23 kg/m^2(37)^. Similarly, in another study, South Asian adults in the highest tertile of visceral adipose tissue had a lower 25(OH)D than those in the lowest tertile of visceral adipose tissue^(30)^. Adiposity in the South Asian population, as in other ethnic groups, is a clear risk factor for vitamin D deficiency and public health approaches are needed to tackle this problem for higher 25(OH)D concentration as well as for preventing and/or treating other important problems such as CVD and type II diabetes.

## Likely health effects of vitamin D deficiency in western South Asians

Nutritional vitamin D deficiency, with or without concurrent calcium deficiency, is a direct cause of osteomalacia in adults and rickets in children. In the UK, there has been recent call for political action to reduce rickets, osteomalacia and infant hypocalcaemia^(61)^. Rickets and infant hypocalcaemia are relatively rare, with updated incidence estimates for hypercalcaemic seizures being reported in children (3⋅49 per million children aged 0–15 years) in the UK and Ireland^(62)^. However, they have a high morbidity and infant hypocalcaemia is potentially fatal. Of note, almost all cases of infant hypocalcaemia^(62)^ and childhood rickets^(63)^ in western countries are of Black or Asian ethnicity, likely due to the known vitamin D deficiency in these populations.

In adults, vitamin D deficiency is associated with osteomalacia as well as generalised bone pain and weakness. Vitamin D deficient South Asian adults are frequently referred to pain clinics^(64)^. A case report in Ireland showed severe proximal myopathy in two lactovegetarian South Asian adults, with symptom resolution following supplementation with calcium and vitamin D^(65)^. Also, a study from the 1970s of South Asians living in Rochdale, UK, found evidence of bone pain, muscular weakness and general pain/weakness in individuals with 25(OH)D at about 25 nmol/l, with tetany observed in those with 25(OH)D at about 12 nmol/l^(7)^. Measurement techniques for 25(OH)D have changed since the 1970s so these values must be viewed with caution, but the study illustrates the point that clinically relevant findings have been shown in South Asians with similar 25(OH)D concentrations to that seen today.

Vitamin D deficiency is associated with increased risk of a variety of other chronic health conditions in addition to musculoskeletal health and may be contributing to known health inequalities in western populations. In addition to the earlier evidence for musculoskeletal conditions there is some evidence that South Asians are more likely to suffer from other health conditions related to vitamin D deficiency. For example, a recent UK-based study found that South Asians had a higher prevalence of respiratory tract infections than did some other ethnic groups, although survival was actually higher^(66)^. In a Canadian study, South Asian men were more likely than White European men to fill prescriptions for antibiotics for all health conditions^(67)^, suggesting a higher burden of infectious disease. Of course, this is observational data and may not be due to vitamin D deficiency alone, although it is known that vitamin D plays a key role in the immune system and that vitamin D supplementation of deficient individuals reduces the risk of respiratory infections^(68)^. Therefore, it is feasible that vitamin D deficiency is making a contribution. Vitamin D deficiency has been associated with a variety of chronic health conditions such as heart disease, diabetes, autoimmune disease and osteoporosis^(1)^ but some of these associations are based on observational data and are not fully proven yet. Of note, CVD and diabetes are already highly prevalent among western dwelling South Asians^(69)^ and vitamin D deficiency could possibly be contributing to the burden of disease for these conditions.

## Future public health interventions to improve 25-hydroxy vitamin D concentration in western dwelling South Asians

### Food fortification

There have been recent calls for food fortification programmes in many countries to tackle the problem of vitamin D deficiency. A recent paper argues particularly for the need for mandatory food fortification and monitored antenatal and infant supplementation, to tackle vitamin D deficiency in UK dwelling Black and South Asian individuals^(61)^. This is on the basis of the clear need to prevent childhood rickets, adult osteomalacia and infant hypocalcaemia in this population^(61)^.

In terms of the form of vitamin D used in food fortification, there is recent evidence, from an intervention trial and a meta-analysis, to suggest that vitamin D_3_ is more effective than vitamin D_2_ in raising 25(OH)D concentration, in both South Asians and White Europeans^(70,71)^. However, this may cause issues for the South Asian population. Vitamin D_3_ is usually obtained from the lanolin in sheep's wool and so there may be concern that (1) it is not a vegan product (for vegan South Asians) and (2) that it may not be labelled by the manufacturer as Halal compliant, so Muslim South Asians may not feel happy to consume it. There is a form of vegan vitamin D_3_ which is now commercially available which is extracted from Norwegian lichen. However, it is currently very expensive, prohibiting its use in widespread fortification. Therefore, any current food fortification for South Asians would likely need to use vitamin D_2_.

Food fortification suitable for South Asians will also need to be in the correct vehicle for biological effectiveness, as well as being a commonly consumed food by South Asians. Milk fortification nationally has been highly successful in improving vitamin D status in Finland^(72)^ but this may not be suitable for South Asians as many are lactose intolerant so do not consume milk products in significant amounts. Suitable vehicles may include another drink such as orange juice or a sweet or savoury snack item (e.g. biscuit). Indeed, the intervention trial by Tripkovic *et al*. showed that vitamin D contained in orange juice and a sweet digestive style biscuit were equally effective at raising 25(OH)D concentration in South Asian women, using lipid encapsulated vitamin D^(70)^. This suggests that vitamin D does not need to be added to a high fat food product if it is correctly encapsulated and that it is still absorbed just as well as from a low fat food product. Similarly, in Denmark, Gronborg *et al*. found that 20 μg daily, given as cheese, eggs, fortified yoghurt or crisp bread, was successful in raising 25(OH)D concentration to 54 nmol/l in Pakistani women, with only 3 % having 25(OH)D <30 nmol/l, compared with 34 % in the control group^(24)^. This suggests that a wide variety of food products are suitable vehicles for vitamin D fortification.

Alternatives to a national food fortification programme include helping individuals to increase their intake of commercially available vitamin D bio-fortified foods, some of which may be dietary compliant foods for South Asians. These foods typically involve the addition of vitamin D to an animal's food (e.g. for meat or eggs), or irradiating a product directly with sunlight (e.g. mushrooms). Of note, UVB-irradiated mushrooms provide a relatively higher vitamin D intake per gram than normal mushrooms, but still may need to be eaten in large quantities as they are still relatively low in vitamin D content. However, these products may be more expensive than non-biofortified alternatives and may not be widely available in all shops, meaning it may not reach poorer households, or households with less access to larger supermarkets or specialist shops.

### Increasing supplement use

Research has shown that supplement use is a feasible way to raise 25(OH)D concentration in western dwelling South Asians, although the doses used in research studies may be higher than the recommended vitamin D intake limits in some countries so lower doses may be needed for routine, long term use. Observational studies suggest a benefit of vitamin D supplement use on 25(OH)D concentration. In a cross-sectional study of young Canadian adults, a supplement of 20 μg daily was associated with only 2 % of the sample having a 25(OH)D concentration <50 nmol/l, compared with 39 % of those who took <20 μg daily^(47)^. Of note, 55 % of those not taking a supplement at all had a 25(OH)D measurement <50 nmol/l, compared with only 8 % of those who took a supplement^(47)^. Of course in these observational studies there could be other confounders influencing supplement intake which also directly, or indirectly, influence vitamin D status (e.g. BMI and socioeconomic status).

Evidence from randomised control trials confirms that commonly consumed supplement intakes are effective at raising 25(OH)D concentrations in South Asians. In a New Zealand-based study, supplementation with 100 μg daily was successful in bringing South Asian women (aged 21 years and above) from a baseline average of 21 up to 75 nmol/l, with 75 % of individuals being 55 nmol/l or above^(73)^. A 1-year Danish vitamin D supplementation trial showed that 20 μg daily raised median 25(OH)D concentration 4-fold in Pakistani women, to about 46 nmol/l, with 75 % of women at 39 nmol/l or greater^(74)^. Similarly, 25(OH)D concentration increased 3-fold in Pakistani men to about 55 nmol/l with 75 % of men at 45 nmol/l or greater^(74)^. Twenty micrograms daily is the current US Institute of Medicine recommended intake for people aged over 70 years^(40)^ so could in fact be a useful starting point for discussion of a new vitamin D intake guideline for South Asians.

However, there are issues with the use of supplementation. Universal supplementation is not a popular strategy for policy makers or the general public. It is problematic if individuals do not want to take tablets at all, or if they forget to take them. Annual bolus doses of vitamin D, although convenient and can be administered by healthcare providers at the same time as annual vaccinations (e.g. influenza), so overcome the compliance problem, have been found to be potentially detrimental to bone health^(75)^ and cannot currently be recommended. Importantly, the qualitative research discussed previously in this review^(55,56)^ emphasises that South Asians are often willing to take oral supplements (or even injected supplements) in principle. However, as discussed earlier, of course there may be issues with the formulations of the supplement (e.g. potentially needing to be vegan or Halal) which were not explored in these studies.

### Increasing sunlight exposure

Increasing sunlight exposure may be problematic for many South Asian women, particularly Muslim women, who may not be able to expose their arms, or face and arms in public. Also, it is likely that relatively long sun exposures would be required to produce enough vitamin D. For example, as demonstrated in the paper by Farrar *et al*. which assessed the impact of artificial UVB from a whole body irradiation cabinet on 25(OH)D concentration in South Asian women wearing shorts and t-shirt. This study found that short sun exposures (20 standard erythemal doses given over a period of 6 weeks, the equivalent to 15 periods of 30 min in natural summer sunlight) may not be sufficient to increase 25(OH)D concentration to 50 nmol/l^(48)^. Therefore, even if safe sun exposure areas were to be created for South Asian women they would likely need to spend significant amounts of time in these areas to get the vitamin D benefit.

In terms of visits abroad, it is known that sun holidays are predictive of higher 25(OH)D concentration^(76,77)^. Specifically, in terms of western dwelling South Asians, observational research does suggest that visits to the Middle East or Asia might contribute significantly to improving 25(OH)D concentration. For example, in the 1970s/1980s in Rochdale (UK), in a sample of South Asian Islamic adults, a visit in the past 6 months to Pakistan or the Middle East was associated with a higher 25(OH)D (32 nmol/l), a clinically meaningful increase compared with those who stayed in the UK^(7)^. This study was undertaken 40 years ago but the results are likely to still be applicable today. However, many South Asian families cannot afford to travel abroad, particularly those from the poorest households. Recent research suggests that those in the highest quintile of socioeconomic status have the highest 25(OH)D concentration^(78)^, suggesting that South Asians from the lower socioeconomic status groups may be at risk of lower 25(OH)D concentration. Moreover, it may be that those who can afford a visit abroad are likely to be richer, and so the positive association between 25(OH)D concentration and holidays may be slightly confounded (e.g. with other factors that might increase 25(OH)D concentration, such as gardening as a hobby^(79)^).

### A need for a South Asian specific dietary intake for vitamin D?

Public health bodies could now consider producing a South Asian specific dietary guideline for vitamin D. The current recommended intake in the UK is 10 μg daily for those aged 4 years and over, which under the Holick formula of 2⋅5 μg raising 25(OH)D concentration by 2⋅5 nmol/l^(80)^, would raise 25(OHD) concentration by only 10 nmol/l. This is unlikely to get many South Asians anywhere near the 50 nmol/l optimal level set by the US Institute of Medicine (2011) although it would help many South Asians meet the 25 nmol/l level, recommended to avoid musculoskeletal health problems, set by the UK Scientific Advisory Committee on Nutrition^(42)^. One starting point could be to assess the efficacy and safety of recommending 20 μg daily for all South Asian adults, as already recommended by the US Institute of Medicine^(40)^ for people over 70 years of age. Using the Holick formula^(80)^, 20 μg daily would be predicted to increase 25(OH)D concentration by 20 nmol/l and would bring all South Asian adults above the 25 nmol/l level and many towards the 50 nmol/l level. Of course, a thorough risk assessment would need to be done to ensure safety of this intake across the South Asian adult population. A revised guideline may also be useful for Black populations who also have significant vitamin D deficiency.

Of course, the problem still exists in terms of how to help South Asian adults to achieve this intake, particularly when even the current guidelines (e.g. 10 μg in the UK) are difficult to achieve from diet alone, and as discussed previously in this review, current South Asian intakes are generally only about 1–4 μg daily depending on the region of residence and specific South Asian group.

## Conclusion

Vitamin D deficiency is at epidemic proportions in western dwelling South Asians, particularly those living in Europe. The majority of research has been conducted in the UK, mainland Europe and Canada. There has been little research into the 25(OH)D concentration of South Asians living in the USA, Australasia and South Africa. Reasons for vitamin D deficiency include low vitamin D intake from food and supplements, a covered dress style as well as little sun exposure and relatively high levels of adiposity. Adverse health effects of vitamin D deficiency can include childhood rickets, infant hypocalcaemia and adult osteomalacia. A wide range of chronic diseases may also be more common in those with vitamin D deficiency. Future public health interventions may include more widespread food fortification of vitamin D in foods commonly consumed by South Asians, as well as increasing consumption of natural sources of vitamin D or newly developed bio fortified foods. Another dietary option is to increase vitamin D containing supplement use. Safe ways of increasing sun exposure need to be developed, including potentially the use of safe spaces designed for women to be able to expose skin to the sun. A South Asian specific guideline for vitamin D intake may be required, with 20 μg daily being a potentially effective option, but culturally appropriate measures would be needed to achieve this through the aforementioned listed approaches.
